# Risk-based management of drinking water safety in Australia: Implementation of health based targets to determine water treatment requirements and identification of pathogen surrogates for validation of conventional filtration

**DOI:** 10.1016/j.fawpar.2017.08.002

**Published:** 2017-08-30

**Authors:** Paul Monis, Melody Lau, Martin Harris, David Cook, Mary Drikas

**Affiliations:** Australian Water Quality Centre, South Australian Water Corporation, GPO Box 1751, Adelaide, SA 5001, Australia

**Keywords:** *Cryptosporidium*, Surrogate, HBT, Water, Conventional filtration

## Abstract

The safety of drinking water in Australia is ensured using a risk management framework embedded within the Australian Drinking Water Guidelines (ADWG). This framework includes elements for hazard identification, risk assessment, risk mitigation, verification of barrier performance and monitoring for any changes to the hazards that influence source water quality. The next revision of the ADWG will incorporate Health-Based Targets (HBTs) for achieving microbiologically safe drinking water. This incorporates Quantitative Microbial Risk Assessment and the metric of Disability Adjusted Life Year (DALY) to define safety, with a target of 1 × 10^− 6^ Disability Adjusted Life Year (1 microDALY) set as the maximum tolerable disease burden from drinking water, which in the case of *Cryptosporidium* is < 1.3 × 10^− 5^ oocysts/L. The resulting product water specification, in combination with knowledge of pathogen challenges in source waters, allows the determination of the treatment requirements to ensure public safety. The ADWG revision provides default removal values for *Cryptosporidium* for particular treatment processes, such as conventional coagulation and dual media filtration. However, these values are based on assumptions regarding treatment plant design, operation and water quality. To properly manage risk and demonstrate compliance with the guidelines, water utilities may need to validate treatment performance for *Cryptosporidium* removal. A particular limitation is the absence of *Cryptosporidium* surrogates for full-scale filter validation. This paper will provide an overview of risk-based management of drinking water safety in Australia, the development of health-based targets for microbial pathogens and the evaluation of *Cryptosporidium* surrogates for conventional coagulation and dual media filtration.

## Highlights

• Used pilot-scale coagulation, sedimentation, granular media filter water treatment
• Compared the removals of *Cryptosporidium* oocysts and surrogates
• Pilot-scale treated water quality was comparable to full-scale treatment.
• Modified microspheres most similar to oocyst filtration removal
• *Clostridium* spores, algae and turbidity conservative indicators of oocyst removal
• Turbidity, algae have great potential as on-line indicators for oocyst removal.

## Graphical abstract

**Figure:**
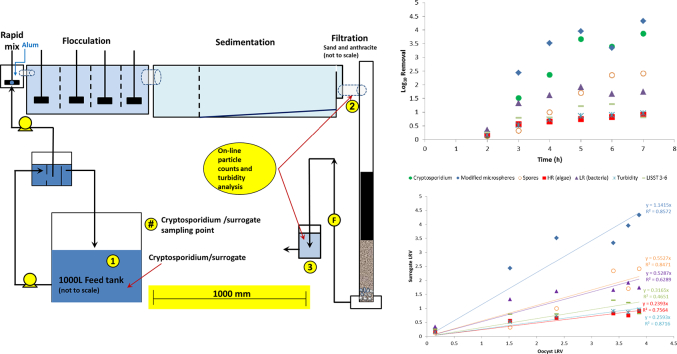

## 1 Introduction

### 1.1 Approaches for managing drinking water quality

The World Health Organisation (WHO) has identified access to safe drinking water as a basic human right (WHO, 2011). In combination with adequate sanitation and hygiene, safe drinking water provides significant health benefits to the community, improving quality of life and reducing the social and economic burden of disease (WHO, 2011). Achievement of these goals varies across the world, with local success influenced by a variety of environmental, economic, social and geopolitical factors. Different approaches can be used to ensure the safety of drinking water. The WHO provides a risk-based framework for managing the safety of drinking water. This framework has key components: using public health outcomes to define tolerable levels of contaminants in drinking water (health-based targets); the use of water safety plans incorporating hazard identification and control measures, monitoring of processes or barrier performance; implementation of management systems including documentation and communication; and review and audit of water safety plans (WHO, 2011). The WHO drinking water guidelines are comprehensive, also providing information regarding contaminants (microbial, chemical and radiological including guidance values), treatment options, disinfection and analytical methods (WHO, 2011).

In the United States of America (USA), drinking water safety is federally legislated through the Safe Drinking Water Act, which allows the United States Environmental Protection Agency to implement the provisions within the act and to regulate the quality of drinking water to protect public health. The approach used in the USA is prescriptive, with the National Primary Drinking Water Regulations used to define maximum contaminant loads or treatment requirements to provide legal protection of public health from drinking water contaminants (USEPA, 2013). These regulations are further supported by rules that mandate treatment techniques and/or monitoring requirements for chemical or microbial contaminants. For example, the Long Term 2 Enhanced Surface Water Treatment Rule prescribes the analytical methods and treatment requirements to protect public water supplies from contamination by the enteric pathogen *Cryptosporidium* (USEPA, 2006).

The methodology used to manage the safety of drinking water in Australia uses the same philosophy as the WHO guidelines, providing a risk management framework rather than prescribing monitoring requirements, analytical methodologies or treatment processes for meeting regulated water quality targets. The Australian Drinking Water Guidelines (ADWG) incorporates aspects from standards for quality systems (ISO 9001), risk management (AS/NZS 4360:2004) and the management of food safety (hazard analysis and critical control points (HACCP)). The ADWG recognises the amount and complexity of the information presented and so it clearly articulates the fundamental guiding principles (such as “The greatest risks to consumers of drinking water are pathogenic microorganisms. Protection of water sources and treatment are of paramount importance and must never be compromised.”) and the structure of the framework for the management of drinking water quality (NHMRC, 2016b). The management framework endorsed by the ADWG has 12 elements, broadly grouped as a commitment to drinking water quality management, systems analysis and management (effectively the HACCP component of the framework), supporting requirements (including elements of quality systems such as staff training, document control) and review (NHMRC, 2016b). There is no Australian federal legislation covering drinking water safety and the ADWG is a guideline for best practice, rather than a standard, so by itself is not enforceable. Instead, the Australian states and territories separately legislate to ensure drinking water safety, typically via acts or regulations that specify, among other licensing requirements, that water suppliers must comply with the ADWG.

### 1.2 Health-based targets for managing contaminants in water

One approach to managing contaminants present in drinking water sources is the application of health-based targets (HBTs). The WHO describes four types of health-based targets: health outcome targets, water quality targets, performance targets, and specified technology targets (WHO, 2011). These targets are used in combination to achieve safe drinking water. Health outcome targets are defined using concepts such as tolerable disease burden (less than a particular frequency of disease from drinking water) in the case of microbiological contaminants, or no adverse health effects in the case of chemical or radiological contaminants. Provided that sufficient toxicological data are available, the derivation of no adverse effect levels for chemicals is relatively straightforward. There are different approaches to defining tolerable disease burden; in the USA this is based on the frequency of a particular disease occurring, whereas the WHO also incorporates disease severity to calculate disability-adjusted life years (DALY), which is effectively the average decrease in life expectancy as a consequence of a person contracting a particular disease. Water quality targets are guideline values that are applied to drinking water to meet health outcome targets. These are generally only used for chemicals, since the guideline values for most chemicals are usually at levels that can be readily measured. In contrast, it is neither practical nor technically possible to measure the extremely low levels of microbial contaminants required to demonstrate compliance with health outcome targets. Instead, performance targets are used to define the amount of source water treatment required to reduce the number of pathogens in drinking water so that the health outcome target is not exceeded. These performance targets can be achieved using specified technologies, which have been validated to remove particular contaminants of concern.

In Australia, the ADWG provides guideline values for chemical contaminants but not for microbial contaminants. The draft revision of the ADWG incorporates microbial HBTs and was released for public comment in 2016 (NHMRC, 2016a). This revision uses 1 micro DALY as the health outcome target for waterborne pathogens, describes the use of *Escherichia coli* or specific pathogen monitoring data for risk-based categorisation of water sources, suggests minimum treatment targets (log_10_ removal values (LRV)) for protozoa (*Cryptosporidium*), bacteria, and viruses for each risk category, and provides default LRV treatment credits for commonly used treatment processes (NHMRC, 2016a). The draft microbial HBT framework also makes provision for the validation of treatment processes that do not have well established published LRVs or for when credit is sought for a process above the default LRV specified in the framework.

### 1.3 Significance and management of *Cryptosporidium* in water

The enteric pathogenic protozoan *Cryptosporidium*, which has a low infectious dose, limited options for effective drug treatment, and resistance to the levels of chlorine used for disinfecting drinking water (Fayer, 2004), is recognised as a high risk to public health and has been a particular focus for the implementation of the HBT framework in Australia. While default LRVs for *Cryptosporidium* have been provided for common treatment processes (e.g. combined coagulation, flocculation, and sedimentation, followed by granular media filtration), these values assume “Good Practice Operation” of optimally designed and maintained water treatment plants and the filtration processes are reliant on the use of product water turbidity to determine the LRV credit (NHMRC, 2016a). Ideally, water treatment plants at high risk of *Cryptosporidium* challenge would have their performance validated. In practice, it is not feasible to conduct a full-scale validation using *Cryptosporidium* oocysts, in part due to safety considerations but also due to analytical costs and the difficulty in obtaining sufficient quantities of oocysts for full-scale testing. An alternative is to use a surrogate, ideally a parameter that is naturally present and can be measured continuously to ensure that performance targets are being met.

A number of surrogates have been reported for *Cryptosporidium* oocysts, including naturally occurring microorganisms, particulates naturally present in source water, and artificially produced particles (microspheres). Naturally occurring microorganisms include aerobic spore-forming bacteria (Mazoua and Chauveheid, 2005, Nieminski and Bellamy, 2000), sulphite-reducing clostridia spores (Galofre et al., 2004, Hijnen et al., 2004), and the green algae *Chlorella* (Hsu, 2006). Artificial surrogates include carboxylated microspheres (Dai and Hozalski, 2003), fluorescently-labelled microspheres (Amburgey et al., 2005), and glycoprotein-coated microspheres (Pang et al., 2012). All of these surrogates are in a similar size range compared with oocysts, with the glycoprotein-coated microspheres also possessing a similar surface charge to oocysts. However, few studies have directly compared the removal of oocysts and the proposed surrogates, and those that have conducted comparisons have focussed on demonstrating removal equivalence for a narrow range of treatment conditions.

The turbidity of surface waters often correlates with oocyst abundance (Health Canada, 2012, Rose et al., 1991). The removal of turbidity by conventional water treatment has been shown to correlate with oocyst removal (LeChevallier et al., 1991, Nieminski, 1992). Based on filtration studies, filtered water achieving a turbidity of < 0.1 NTU is taken as being low risk for *Cryptosporidium* (Edzwald and Kelley, 1998, Nieminski and Ongerth, 1995) and such turbidity targets have been incorporated into guidelines (Health Canada, 2012, NHMRC, 2016b, USEPA, 2006, WHO, 2011). However, the endpoint turbidity does not provide direct measurement of filtration efficiency and does not indicate the level of oocyst removal that may be achieved. The removal of particles measured using particle counters has also been reported to correlate with oocyst removal (Dugan et al., 2001, Huck et al., 2002, LeChevallier and Norton, 1992, Li et al., 1997), although this has not been used for full-scale validation of treatment plants and does not appear to have been adopted as a surrogate.

### 1.4 Need for research

Based on the available literature, there is a lack of information comparing the simultaneous removal of oocysts and surrogates under optimal or sub-optimal conventional water treatment conditions, which is required to demonstrate that surrogates can be predictors of oocyst behaviour and used for the validation of treatment performance. This paper reports a preliminary study using a pilot-scale conventional water treatment train to compare the removal efficiency of *Cryptosporidium parvum* oocysts with the removal of a potential oocyst surrogates under optimal and sub-optimal coagulation conditions.

## 2 Materials and methods

### 2.1 Source water

Untreated surface water was obtained from the inlet of the Happy Valley Reservoir (Adelaide, South Australia, Australia) on the 21st March 2016 for jar tests and on the 5th May 2016 for pilot plant tests. Key water parameters, namely dissolved organic carbon (DOC), UV absorbance (UVA) at 254 nm, colour, turbidity and pH were measured using standard or published methods (APHA, 1998, Bennett and Drikas, 1993).

### 2.2 Organisms, surrogates and analytical methods

#### 2.2.1 *Cryptosporidium parvum*

Gamma-irradiated *C. parvum* (Iowa strain) oocysts were purchased from BTF (North Ryde, Australia). Water samples (1 mL) or control oocyst suspensions were serially diluted in sterile phosphate buffered saline (PBS) and enumerated using EasyStain (BTF) and fluorescence microscopy as previously described (King et al., 2017). If oocyst counts were < 1 oocyst/mL, then 10–1000 mL of sample was concentrated by centrifugation at 1800*g* for 20 min, followed by aspiration down to 1 mL and resuspension of the pelleted oocysts prior to enumeration.

#### 2.2.2 Glycoprotein-coated microspheres

Glycoprotein-coated carboxylated microspheres (hereafter termed modified microspheres) were prepared by the Institute of Environmental Science and Research Ltd., Christchurch, NZ, following a previously described method (Pang et al., 2012). Briefly, carboxylated 4.5 μm microspheres were purchased from Polysciences (Warrington, USA) and human glycoprotein was coupled to the microspheres using 1-ethyl-3-(3-dimethylaminopropyl) carbodiimide hydrochloride as a cross-linker. The modified microspheres were stored at 4 °C in PBS containing 0.05% bovine serum albumin. Modified microspheres were enumerated in parallel with oocysts using fluorescence microscopy. The modified microspheres were fluorescent under blue (U-MWB) and UV (U-MWU) light sources and could be readily distinguished from the *Cryptosporidium* oocysts. When required, 10–1000 mL of sample was concentrated by centrifugation at 1800*g* for 20 min, followed by aspiration down to 1 mL and resuspension of the pelleted microspheres prior to enumeration as described above.

#### 2.2.3 *Clostridium sporogenes* spores

*Clostridium sporogenes* NCTC 12935 (BioBall Single Shot catalogue number 413848) were purchased from BTF and initially anaerobically cultured on Columbia Horse Blood Agar (Micromedia, Vic, Australia) for 2 days at 35 °C. A single colony was inoculated into 10 mL of Cooked Meat Broth (Micromedia, Vic, Australia) and incubated anaerobically at 35 °C for 7 days. The culture was heat shocked at 70 °C for 15 min and anaerobically cultured for a further 4–5 days at 35 °C. The spores/bacterial cells in the broth supernatant were transferred to a fresh 10 mL tube and concentrated by centrifugation at 2200 rcf for 20 min (with the brake off). The resulting pellet was washed twice by resuspension in PBS and concentration by centrifugation. After washing, the pellet was resuspended in 10 mL of PBS containing lysozyme (500 μg/mL) and incubated at 37 °C for 2 h. Following lysozyme treatment the spores were pelleted by centrifugation and washed twice using PBS as described above. The spores were resuspended in 11 mL of PBS, 1 mL was removed to enumerate the suspension and the remainder was stored at 4 °C. Prior to use, the spore solution was well mixed and the aliquot to be used in experiments was heat treated at 70 °C for 20 min to ensure that any vegetative cells present were inactivated. For enumeration, samples were serially diluted in PBS, with 100 mL of neat or diluted sample filtered through a 47 mm diameter 0.45 μm membrane and cultured anaerobically on Tryptose Sulfite Cycloserine agar plates (Micromedia, Vic, Australia) at 35 °C for 48 h.

#### 2.2.4 *Chlorella vulgaris*

A culture of *Chlorella vulgaris* was kindly provided by Felicity Roddick (Royal Melbourne Institute of Technology). *Chlorella* were cultured in 75 cm^2^ cell culture flasks using ASM-1 medium (Gorham et al., 1964) at 20–22 °C in a Thermolyne incubator (TRIL-495-1-SD, Thermolyne Scientific, Australia,) under 30 μmol photons m^− 2^ s^− 1^ with a 12 h light/dark cycle. Cells were enumerated by flow cytometry prior to use in experiments. While it was possible to enumerate cells by fluorescence microscopy and flow cytometry, the high background level of other algae in the Happy Valley Reservoir water precluded the enumeration of *Chlorella* added to the reservoir water. Total algae were enumerated using flow cytometry (described below) assuming autofluorescent red particles (indicating the presence of chlorophyll) represented algal cells.

#### 2.2.5 Flow cytometry

An Accuri flow cytometer (nominal detection limit of 1 event/μL without any sample concentration) with a C6 automatic sampling attachment (BD Biosciences) was used for the initial characterisation of oocysts, modified microspheres, spores and algae. While flow cytometry could be used to readily discriminate and count oocysts and the candidate surrogates suspended in ultrapure water (data not shown), the number of particulates present in the source water prevented this technique from being used for direct enumeration (data not shown). In addition, coagulation with alum was noted to induce an increase in the green fluorescence of some particles (data not shown) and so measurement of green fluorescence was not used as a parameter for characterisation of samples. Samples were analysed using default instrument settings for threshold, flow rate and core size, with sample data collected for all available channels (FSC – forward scattered, SSC – side scatter, FL1 (533/30 nm), FL2 (584/40 nm), FL3 (> 670 nm), FL4 (675/25 nm)). Data were analysed using the BD Accuri C6 Analysis software (Accuri Cytometers Inc. version 1.0.264.21), with data visualised using dot plots and histograms. For assessment of algae as a surrogate, algal numbers were estimated using a histogram plot of FL4 signal, with vertical regions drawn at approx. 10^4^ FL4 units to count particles with low or high red fluorescence. It was assumed that events with high red fluorescence represent algae and events with low red fluorescence represent bacteria and other particles.

#### 2.2.6 Particle size distribution analysis

Particle size distribution analyses were conducted on grab samples using a LISST-Portable XR instrument (Sequoia, USA), which can count particles in the 0.37 μm–460.27 μm size range. Data were collected using the Mie optical model for polystyrene beads, following the manufacturer's instruction for operating the instrument. The LISST measures the volume of each size class per volume of sample (μL/L). This output was converted to a particle count (particles/L) by calculating the volume of a particle in each size class (assuming a sphere and using 4/3πr^2^) and dividing the volume of particles for a given size by the volume calculated for that particle size. Modified microspheres of known size (Polysciences Inc) were used to check the accuracy of the particle size analysis (data not shown). On-line total particle counts from the pilot plant were measured using Liquilaz E20 (2–125 μm particles) and S05 (0.5–20 μm particles) instruments (Particle measuring systems, USA) for settled water and filtered water respectively. For the purposes of calculating coagulation sedimentation LRVs for the pilot-scale testing, the settled water without coagulant was used as the raw water particle measurement there was no direct on-line measurement option for the raw water).

#### 2.2.7 Turbidity

The turbidity of settled and filtered water from the pilot plant was measured continuously using a 1720E low level turbidity meter (HACH, USA). Grab samples from jar testing and pilot plant experiments were analysed using a 2100AN bench turbidity meter (HACH, USA).

#### 2.2.8 Calculation of log_10_ removal values

In general terms, the log_10_ removal values (LRVs) were calculated by first log_10_ transforming the measured oocyst or surrogate values and then subtracting the post-treatment value from the pre-treatment value. Coagulation LRV was calculated using: log_10_(untreated water value) − log_10_(settled water value). Filtration LRV was calculated using: log_10_(settled water value) − log_10_(filtered water value).

### 2.3 Jar testing

A jar test experiment was conducted to evaluate the effect of the coagulant doses on oocyst and surrogate removal rates and also to ensure that the analytical methods provided satisfactory performance on spiked samples. After spiking with oocysts and surrogates the water was subjected to jar testing using a programmable 6-paddle stirrer with gator jars (Phipps and Bird, USA). Following addition of alum (0, 10 or 30 mg/L) the water was flash mixed for 1 min at 200 rpm, followed by 14 min slow mixing at 20 rpm and settling for 15 min. Samples were collected prior to coagulation, after clarification and after filtration through Whatman No. 1 filter paper with a rated porosity of 11 μm.

### 2.4 Pilot plant experiments

**Fig. 1:**
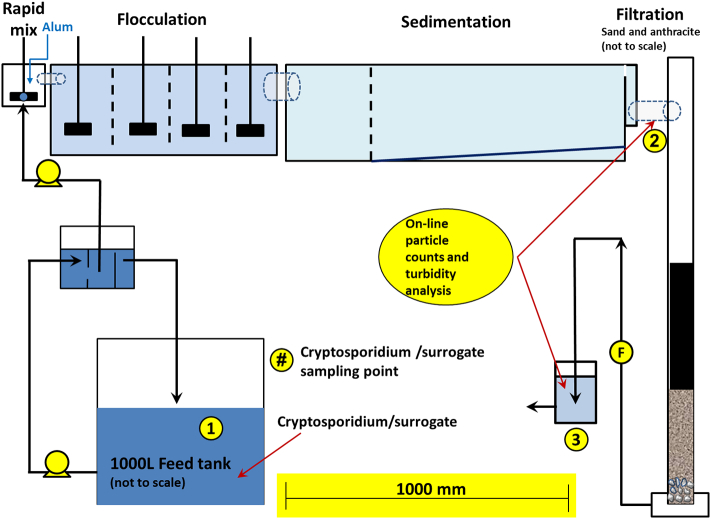
Pilot plant schematic. Numbers indicate sampling locations for untreated water (1), settled water (2) and filtered water (3).

The layout of the pilot plant, including sample collection points, is shown in Fig. 1. The system was designed to provide the same treatment process steps as the full-scale Happy Valley water treatment plant. The dimensions of the flash mixing chamber, flocculation bays and sedimentation tank are given in Table 1. The filter had a radius of 0.095 m, with a bed depth of 1.2 m, a surface area of 0.0071 m^2^ and an empty bed contact time of 3.8 min. The filtration rate was 15.2 m/hr., at a flow rate of 0.108 m^3^/hr. The media depths were Anthracite 0.65 m, Sand 0.35 m, and Gravel 0.2 m. The filter contained 0.6 mm filter sand (product number 7C, River Sands, Carbrook, Queensland) and anthracite (filter coal, James Cumming and Son, Auburn, New South Wales). *Cryptosporidium* oocysts and surrogate particles/organisms were added to a bulky bin containing approximately 1000 L of Happy Valley reservoir water. The target final concentrations after dosing were 10^2^ oocysts/mL, 10^2^ modified microspheres/mL, 10^3 −^ 10^4^ spores/mL and 10^3^ *Chlorella*/mL. These concentrations were constrained by the numbers of stock organism/surrogate available. The selected volume was sufficient to operate the pilot plant for 7–8 h for filter trials, at a flow rate of 1.8 L/min, with rapid mixing of 17–140 s, a flocculation time of 22 min, a sedimentation time of 56 min and a filtration rate of 15 m/hr. The turbidity of the settled water and filtered water was measured on-line at 15 min intervals. These same sample points were also subjected to on-line particle analysis in 1 min intervals. Experiments were conducted at ambient temperature. Alum doses started at 0 mg/L and were sequentially increased to 15, 30 or 40 mg/L at 2, 4 and 6 h into the filter run. The purpose of using a range of alum doses was to allow measurement oocyst and surrogate removal at different levels of coagulation and filtration performance. Coagulant aid was not used for these experiments. Grab samples were collected hourly from the inlet, settled water and filtered water locations as shown in Fig. 1.

**Table 1:** Dimensions and volumes of flash mix, flocculation and sedimentation stages.

Process	Dimensions (m)				Volume (L)
	Length	Width	Height	Water height	
Flash mix	0.1	0.1	0.15	0.07	0.7
Flocculators × 3 in series	0.15	0.15	0.2	0.16	3.6
Sedimentation	0.6	0.2	0.3	0.23	27.6

## 3 Results

**Fig. 2:**
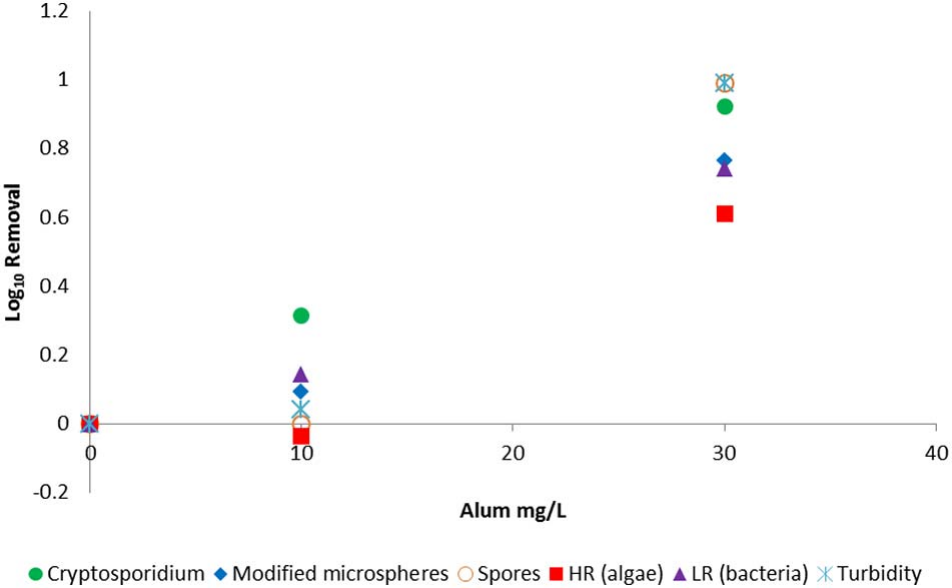
Graph comparing log_10_ removal values for *Cryptosporidium* oocysts and surrogates following exposure to different coagulant doses and sedimentation in a jar test. Spores are *Clostridium sporogenes*. HR indicates high red fluorescent particles (algae), LR indicates low red fluorescent particles (bacteria and other material). (For interpretation of the references to colour in this figure legend, the reader is referred to the web version of this article.)

**Fig. 3:**
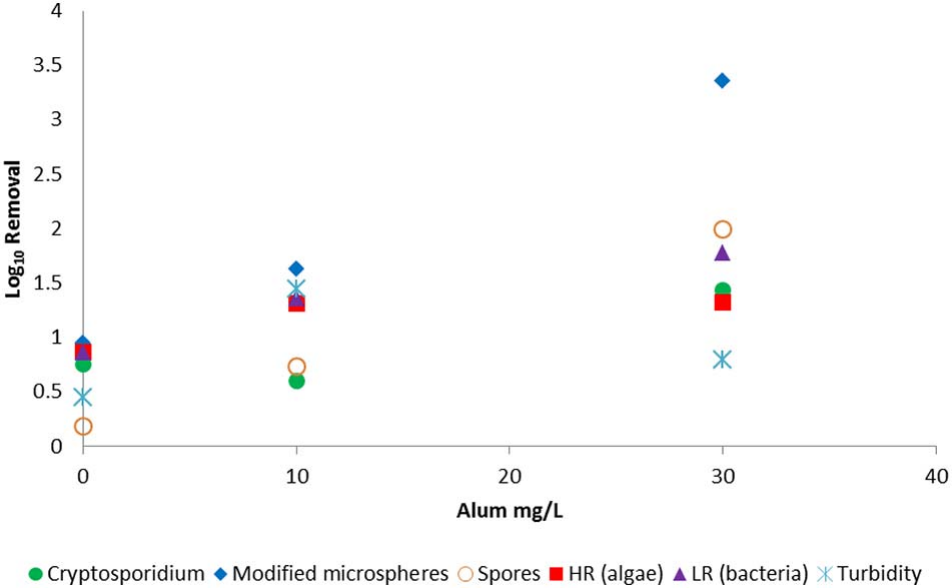
Graph comparing log_10_ removal values for *Cryptosporidium* oocysts and surrogates following exposure to different coagulant doses, sedimentation and filtration in a jar test. Spores are *Clostridium sporogenes*. HR indicates high red fluorescent particles (algae), LR indicates low red fluorescent particles (bacteria and other material). The spore and oocyst LRVs for the 30 mg/L alum dosed sample were greater than results (no oocysts or spores detected in the product water for this sample). (For interpretation of the references to colour in this figure legend, the reader is referred to the web version of this article.)

The water quality parameters for the Happy Valley water used in bot jar test and pilot-scale experiments are presented in Table 2. A turbidity measurement for the 21/3/2016 water prior to addition of the spiked material (oocysts and surrogates) was not available. Addition of the spike resulted in a minor increase in turbidity but did not appear to affect other parameters such as DOC or UVA _254 nm_. The jar testing data suggested that for coagulation and sedimentation, the surrogates were conservative indicators of oocyst removal for an alum dose of 10 mg/L, and had slightly lower (modified microspheres, algae, bacteria) or similar (turbidity, spores) removal compared with oocysts for an alum dose of 30 mg/L (Fig. 2). Removals through the filter paper produced more variable results, with the modified microspheres exhibiting a much higher LRV compared to the other surrogates and oocysts (Fig. 3). This may be an artefact for spores and oocysts because the LRVs for these were greater than results (spores and oocysts were below detection limit in the filtered water for the 30 mg/L alum-dosed sample). Filtration was able to remove modified microspheres, oocysts, bacteria and algae (up to 1 LRV) in the absence of coagulation, though turbidity removal was lower (0.5 LRV) and spores were poorly removed. While the coagulation/sedimentation using 10 mg/L alum resulted in low LRVs for the surrogates, the exposure to coagulant appeared to increase filtration LRV for the surrogates.

**Table 2:** Summary of key quality parameters of reservoir water used in jar testing (21/3/2016) and pilot-scale experiments (5/5/2016).

Collection date	Sample description	DOC (mg/L)	UVA _254 nm_ (cm^− 1^)	Colour (HU)	Turbidity (NTU)	pH
21/3/2016	Un-spiked	4.40	0.104	10	4.30	8.0
5/5/2016	Un-spiked	4.60	0.088	8	1.43	7.7
5/5/2016	Spiked	4.60	0.089	8	1.62	7.7

**Fig. 4:**
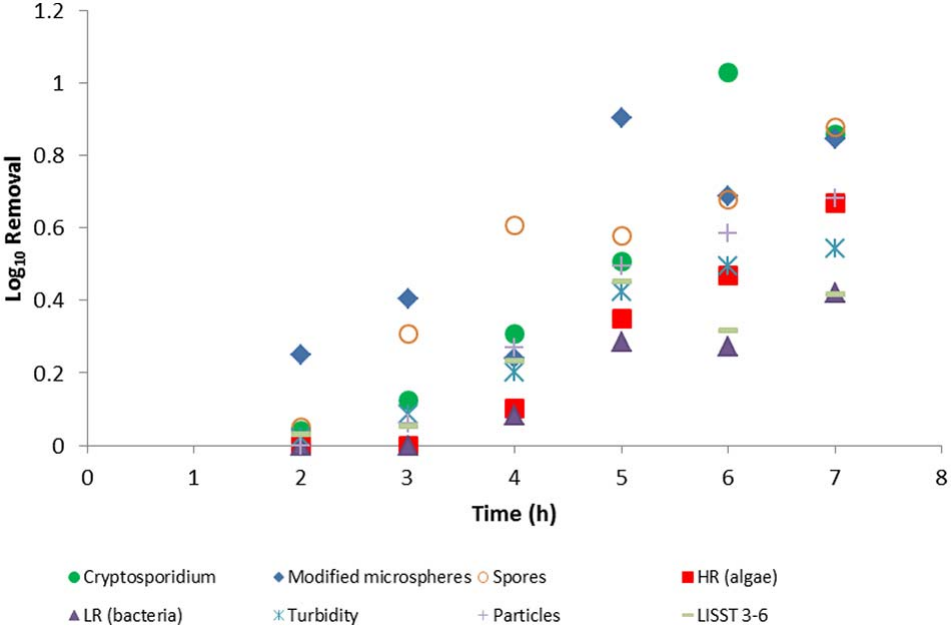
Graph comparing log_10_ removal values for *Cryptosporidium* and surrogates following exposure to different coagulant doses and sedimentation in a pilot-scale test. Spores are *Clostridium sporogenes*. HR indicates high red fluorescent particles (algae), LR indicates low red fluorescent particles (bacteria and other material). Particles represent total particle counts from an on-line particle meter. LISST 3–6 represent particles in the 3–6 μm size range. (For interpretation of the references to colour in this figure legend, the reader is referred to the web version of this article.)

The pilot-scale results for coagulation/sedimentation (Fig. 4) were similar to the jar test results, with the maximum LRVs being in a similar range (0.6–0.8 LRV) for the parameters measured. The pilot-scale LRV results were expressed as a function of time rather than alum dose, due to the nature of the pilot plant which has continuous flow and mixing, complicating estimation of the alum concentrations after changing the alum dose points. Assuming steady state after changing the alum doses, the 3, 5, and 7 h time points should represent alum doses of 15, 30, and 40 mg/L respectively. Overall, the surrogates exhibited similar coagulation/sedimentation removals compared with oocysts over the course of the filter run (Fig. 4).

**Fig. 5:**
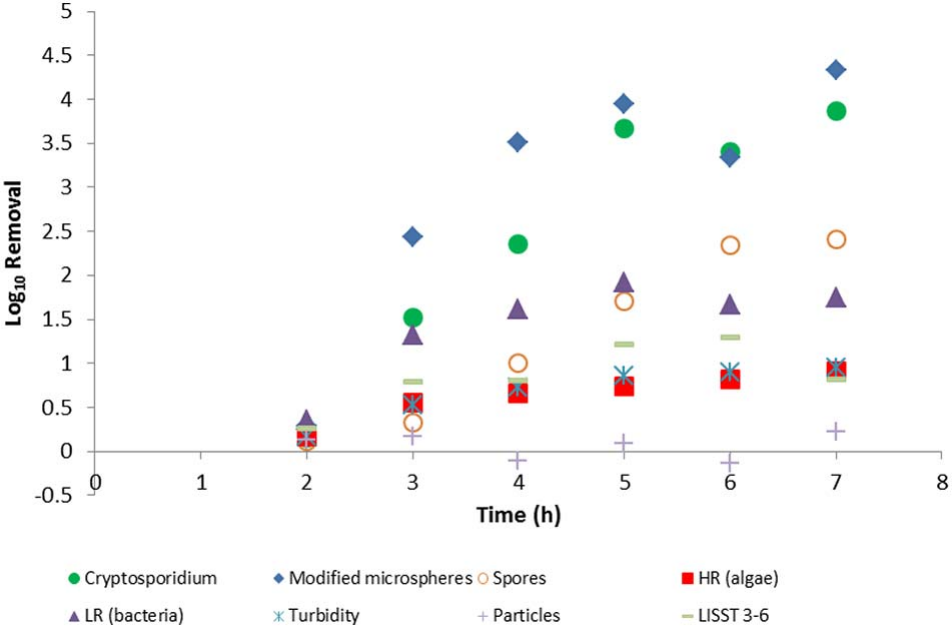
Graph comparing log_10_ removal values for *Cryptosporidium* and surrogates following exposure to different coagulant doses, sedimentation and filtration in a pilot-scale test. Spores are *Clostridium sporogenes*. HR indicates high red fluorescent particles (algae), LR indicates low red fluorescent particles (bacteria and other material). Particles represent total particle counts from an on-line particle meter. LISST 3–6 represent particles in the 3–6 μm size range. (For interpretation of the references to colour in this figure legend, the reader is referred to the web version of this article.)

The removal rates by the pilot-scale granular media filter were different to the filtration using the Whatman paper at low coagulant doses. The removal was low for oocysts (0.15 LRV) and surrogates (0.12–0.35 LRV) at the 2 h time (Fig. 5). Overall the modified microspheres most closely matched the filtration LRVs for oocysts towards the end of the filter run (higher alum doses), but appeared to overestimate oocyst removal during the middle of the filter run. The LRVs for oocysts were much higher than the other surrogates after the 3 h time point. The removals of turbidity, algae, and particles in the 3–6 μm size range, were similar to each other across the filter run (Fig. 5), with the removals of clostridia spores and bacteria (estimated from the flow cytometry data) slightly higher. The on-line particle analysis did not appear to measure any particle removal by filtration. This may reflect release of particulates from the filter bed that is not related to filter breakthrough. It is worth noting that the 3–6 μm particle measurements were from grab samples using the LISST instrument, whereas the online particle removals were calculated from total particle counts for particles 2–125 μm in the settled water and 0.5–20 μm in the filtered water. These size ranges were predicated by the online instruments used for these measurements and the different performance characteristics of these instruments. In brief, the instrument used to measure filtered product water is highly sensitive and cannot measure raw or settled water samples with high particle numbers. Similarly, the instrument used to measure settled water turbidity was selected to measure higher particle concentrations and lacks the sensitivity for reliable analysis of product water. The different method of measurement may also have affected the LRV calculations using the on-line particle data.

The performance of the surrogates as predictors of oocyst removal was examined by pairwise comparison of the pilot-scale LRVs for oocysts with the LRVs for each surrogate. Linear regression forcing the line through the origin was used as the starting model for curve fitting because it is the simplest and this provided a reasonable fit for the data (generally R^2^ values between 0.5 and 0.8), although allowing a non-zero intercept improved the fit in most instances (increasing most R^2^ values to be between 0.8 and 0.9). The ideal surrogate should have the same behaviour as *Cryptosporidium* oocysts or allow prediction of oocyst removal. The value of the slope from the regression equation provides an indication of the behaviour of the surrogate compared with oocysts. A slope of 1 indicates the same LRVs under the same experimental conditions. A slope of > 1 suggests that the surrogate was removed more efficiently compared with oocysts, over-estimating oocyst removal. A slope of < 1 suggests that the surrogate was removed less efficiently compared with oocysts, providing a conservative indicator of oocyst removal.

**Fig. 6:**
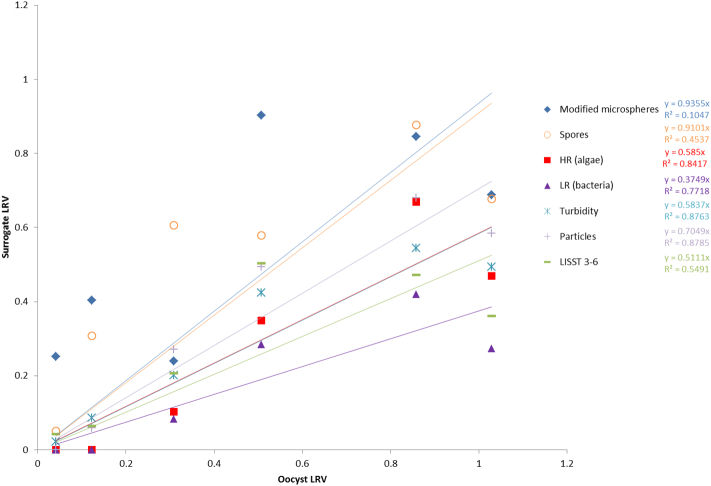
Graph comparing the relationship of surrogate LRV with oocyst LRV for pilot-scale coagulation sedimentation. Spores are *Clostridium sporogenes*. HR indicates high red fluorescent particles (algae), LR indicates low red fluorescent particles (bacteria and other material). Particles represent total particle counts from an on-line particle meter. LISST 3–6 represent particles in the 3–6 μm size range. R^2^ values from linear regressions are indicated to the right of the legend items. (For interpretation of the references to colour in this figure legend, the reader is referred to the web version of this article.)

**Fig. 7:**
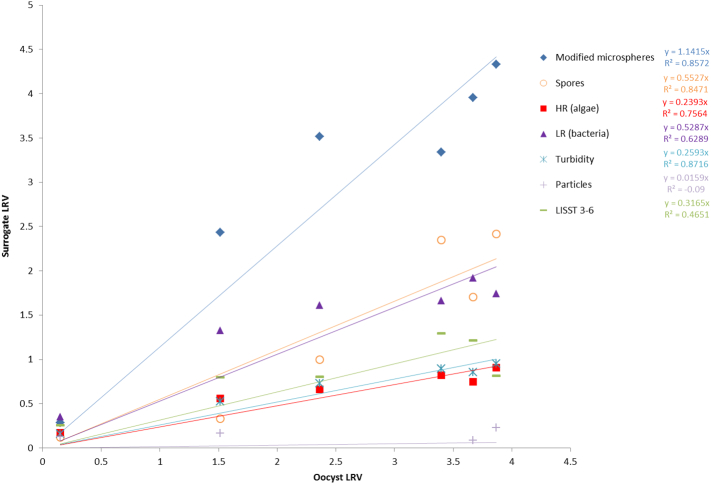
Graph comparing the relationship of surrogate LRV with oocyst LRV for pilot-scale filtration. Spores are *Clostridium sporogenes*. HR indicates high red fluorescent particles (algae), LR indicates low red fluorescent particles (bacteria and other material). Particles represent total particle counts from an on-line particle meter. LISST 3–6 represent particles in the 3–6 μm size range. R^2^ values from linear regressions are indicated to the right of the legend items. (For interpretation of the references to colour in this figure legend, the reader is referred to the web version of this article.)

The results of the regression analyses are shown in Fig. 6, Fig. 7. In the case of coagulation/sedimentation, most of the surrogates were conservative indicators of oocyst removal (slopes between 0.5 and 0.7). The slope for the regression analyses comparing oocyst removal with the removal of spores or modified microspheres was closer to 1, suggesting equivalent removal behaviour of these surrogate and oocysts; however, the R^2^ values for these were low (Fig. 6). Allowing a non-zero intercept increased the R^2^ values to > 0.5 and decreased the slopes to make them comparable with the other surrogates (data not shown). The regression analyses for the filtration LRV data (Fig. 7) provided better goodness of fit compared with the coagulation LRV data. The slopes suggested that modified microspheres exhibited the most similar filtration removal behaviour, although it was slightly higher compared with oocysts (slope > 1). With the exception of on-line particles counts, the other surrogates were conservative indicators of oocyst removal (slopes between 0.24 and 0.55). Of these surrogates, turbidity, spores and algae had high R^2^ values (0.76–0.87).

## 4 Discussion

The validation of treatment processes used to remove *Cryptosporidium* for the production of safe drinking water is of key importance for protecting public health. The absence of suitable surrogates is currently an obstacle to achieving this outcome. Surrogates have been evaluated in the published literature, but in general these studies have not compared the simultaneous removal of the surrogate and oocysts in the same water sample/process. Furthermore, most of the published work has focussed on a direct comparison of filtration curves or % removal performance, and did not determine the removal performance of the surrogate compared to oocysts under a broad range of conditions to evaluate the use of the surrogate as a predictor of oocyst removal. Through the manipulation of coagulation conditions, this is the first study to use a pilot-scale conventional water treatment plant to compare the concurrent removal rates of oocysts and surrogates in the same source water and to evaluate the correlation between oocyst and surrogate removal using regression analysis.

Jar tests with different alum doses were used for the initial comparison of the removal of surrogates and oocysts by coagulation/sedimentation and filtration. The same alum dose range was then used in a pilot-scale conventional coagulation/sedimentation/granular media filtration plant. Perhaps unsurprisingly, the coagulation/sedimentation LRVs for surrogates and oocysts were similar for the jar test and pilot-scale plant when using similar alum doses. Jar testing is a well-accepted method for optimising the coagulant doses used in full-scale water treatment plants. Filter paper was used to simulate filtration for the jar testing and this may be expected to provide different filtration efficiency compared to a granular dual-media filter. This was the case for oocysts, which had a 2 LRV increase in removal in the granular filter compared with filter paper. Similarly, the removal of spores at pilot scale was increased by approximately 0.5 LRV. In contrast, the removal of algae decreased by approximately 0.5 LRV and the removal of modified microspheres and turbidity was relatively unchanged. These removal differences may reflect differences in particle charge after coagulation, filter pore size or transport through the filter.

For the highest alum dose, the filtered water turbidity from jar testing and pilot testing were < 0.1 NTU (data not shown). This is comparable to the performance of the full-scale treatment plant that produces drinking water from the Happy Valley reservoir and below the target of ≤ 0.15 NTU filtered water turbidity to obtain a 4 LRV credit for removal of enteric protozoa as specified within the ADWG draft HBT framework (NHMRC, 2016a). For the pilot-scale testing, filtered water turbidities between 0.05 and 0.09 NTU (data not shown) resulted in *Cryptosporidium* LRVs of 4.2–4.7, which is consistent with the guideline value. Interestingly, filtered water turbidity of 0.19 NTU was achieved at a lower alum dose. While this was near the 0.15 NTU target and just below the ADWG 0.2 NTU target for 3.5 LRV credit for *Cryptosporidium*, this resulted in only 2.7 LRV of oocysts. A similar comparison could not be made for the jar testing because oocyst numbers in the product water were below the limit of detection.

It is difficult to compare the removal efficiencies observed in the pilot-scale experiment reported herein with published studies because few studies expressed results as LRVs or provide sufficient detail of the treatment system or water quality, or used very different filtration processes. A similar pilot-scale study using granular media filters and flocculated water obtained from a full-scale drinking water treatment plant to compare the removal of unmodified microspheres and *Cryptosporidium* oocysts (Amburgey et al., 2005). In that study the oocyst LRVs by filtration were 1.5–1.7, but the coagulant used was ferric chloride and it is not clear how the coagulation efficiency compared with that observed in this study. The LRV for microspheres was approximately 0.5 higher than that observed for oocysts (Amburgey et al., 2005), consistent with the findings in this study. A study using bench-scale filter columns without any apparent coagulation has also reported that spores of *Clostridium perfringens* are conservative indicators of oocyst removal, reporting 2.3–3.2 LRV for spores compared with > 5.3 LRV for oocysts (Hijnen et al., 2004). A similar full-scale study using rapid sand filtration (no coagulation described) reported similar LRVs for spores of *C. perfringens* (3.9) and oocysts (5.3), although as with the previous study, no water quality details were provided (Hijnen et al., 2007). These reported spore removals were higher than those observed in the study for filtration at the highest coagulant dose (though comparable for the combined coagulation/sedimentation/filtration LRV). A different species of *Clostridium* was used in each study, *C. sporogenes* herein and *C. perfringens* in the Hijnen studies.

The regression analyses of the LRV data for coagulation/sedimentation data suggest that all of the tested candidate surrogates have potential use for the validation of oocyst removal by this treatment process. In the case of spores and modified microspheres, the best fit was using a linear equation with a non-zero x-intercept. The data suggest that for these surrogates there is some removal when there is no removal of oocysts, possibly reflecting some sedimentation (or loss through other mechanisms) in the absence of coagulant. The other surrogates all appeared to be conservative indicators of oocyst removal. Of these, turbidity and on-line particle counts are perhaps the most promising, since these can be readily measured on-line and therefore have the potential to be used for continuous prediction of oocyst removal by coagulation/sedimentation. The regression analyses for the filtration LRV data suggested that the modified microspheres most closely matched oocysts in terms of removal behaviour. With the exception of the on-line particle measurement (which showed no removal), all of the other surrogates were conservative indicators of oocyst removal. The apparent poor removal of particles post filtration determined using the on-line particle counters requires further investigation. Different instruments with different sensitivities were used to measure the settled water and filtered water particle counts. These instruments provided total counts for different size ranges of particles and this may have contributed to inaccurate LRV estimates. This possibility is likely considering that the same on-line instrument was used to analyse the raw water and settled water post coagulation and the resulting particle LRVs were similar to the other surrogates. As with the coagulation results, while these indicators were conservative, the majority showed a strong linear correlation with oocyst removal and the goodness of fit of the linear regression analyses suggests that these surrogates could be used to predict oocyst removal. Based on R^2^ values, the two best predictors of oocyst removal for both coagulation/sedimentation and filtration were algae and turbidity. The algal data were derived using flow cytometry counts of particles with high levels of red fluorescence, which does not lend itself to on-line measurement. However, alternative approaches, such as measurement of algal pigments, could be used as alternative to allow on-line measurement of algal removal.

## 5 Conclusions

The results reported herein are only preliminary findings from a single water source and need to be interpreted with caution. Additional pilot-scale and jar test experiments using a wider range of water types and treatment conditions are required to verify these findings. One of the limitations of these types of studies is cost, especially for oocysts and associated analyses when conducted at pilot scale. The similarity of the results for jar tests and the pilot-scale treatment is promising, since if this proves to be more broadly applicable it will allow jar testing to be used to determine the relationships between surrogate and oocyst removal, which would then allow use of the surrogate at full scale. Perhaps the most exciting result is the strong correlations observed between the removals of turbidity and algae with oocyst removal. While they were both conservative indicators of removal, the regression analyses suggest that they could be used to predict oocyst removal. Both of these surrogates are naturally present and can be (or have the potential to be) measured on-line. Further successful validation of these surrogates would allow them to be used to provide continuous monitoring of the performance of conventional water treatment processes for *Cryptosporidium* oocyst removal, enhancing the protection of public health.
